# Pilot Study of Anti-PD-1 Antibody Combined with L-DEP Regimens in the Treatment of Relapsed/Refractory EBV-HLH in Children

**DOI:** 10.1007/s10875-025-01918-7

**Published:** 2025-07-28

**Authors:** Shengchao Wu, Jing Miao, Fenying Zhao, Juan Liang, Xiaojun Xu

**Affiliations:** Division of Hematology-Oncology, Children’s Hospital of Zhejiang University School of Medicine, The Pediatric Leukemia Diagnostic and Therapeutic Technology Research Center of Zhejiang Province, National Clinical Research Center for Child Health, Hangzhou, 310003 China

To the Editor,

Epstein-Barr virus-associated hemophagocytic lymphohistiocytosis (EBV-HLH) is a common subtype of hemophagocytic lymphohistiocytosis (HLH) in children, particularly prevalent among children in East Asia. Although the overall prognosis of EBV-HLH is favorable, some children experience relapses and are difficult to treat, requiring alternative treatment protocols beyond HLH-94/04 (dexamethasone + etoposide ± cyclosporine), such as anti-thymocyte globulin (ATG), CD52 monoclonal antibodies, ruxolitinib, and emapalumab. Previous studies have shown that the L-DEP regimens (PEG-asparaginase combined with liposome doxorubicin, etoposide, and high-dose methylprednisolone) can serve as a safe and effective bridging therapy for hematopoietic stem cell transplantation (HSCT) in both adults and children with refractory and relapsed EBV-HLH (R/R EBV-HLH), achieving an overall response rate of 61.5% and 80% in children with relapsed and refractory HLH and chronic active Epstein-Barr virus infection (CAEBV), but the rate of achieving virological complete response (CR) was only 20% [1, 2]. In recent years, immune checkpoint inhibitors like PD-1 monoclonal antibodies (e.g., Nivolumab, Sintilimab) have been used in the treatment of relapsed and refractory EBV-HLH, showing that anti-PD-1 antibody can restore the T-cell response against EBV [3, 4]. However, using anti-PD-1 antibody during the acute phase of EBV-HLH may exacerbate cytokine storms and pose a risk of fatality [5]. This study aims to further clarify whether the combination of anti-PD-1 antibody and L-DEP regimen can improve the remission rate and enhance the ability to clear EBV in the treatment of R/R EBV-HLH.

From January 2023 to November 2024, this prospective study enrolled 10 children with relapsed and refractory EBV-HLH received L-DEP + sintilimab treatment (liposomal doxorubicin: 25 mg/m² on day 1, etoposide: 100 mg/m² on day 1, methylprednisolone 2 mg/kg on day 1–3 and 0.25 mg/kg on day 4–14, asparaginase: 6000 IU/m² on days 2 and 4, sintilimab 2 mg/kg on day 5). Pediatric patients were classified as having relapsed/refractory HLH if they either: (a) failed to achieve at least a partial response after a minimum of two weeks of therapy with the HLH-94 protocol, or (b) exhibited recurrent disease during or after HLH-94 protocol treatment. There were 5 boys and 5 girls, aged 0.8 to 11.4 years. All children had previously received the HLH-94 regimens, and 3 children had received other treatments (including ruxolitinib, DEP, etc.) when they did not achieved CR after HLH-94 protocol. All these patients had active diseases before L-DEP + sintilimab treatment and had positive serum EBV DNA, with a median level of 1.92 × 10^5^ copies/mL (ranging from 1.19 × 10^3^ copies/mL to 1.2 × 10^6^ copies/mL).

All the patients’ body temperature normalized between the 3rd and 6th day after the initial of this protocol. Significant improvements in key laboratory parameters were observed following PD-1 inhibitor plus L-DEP therapy, particularly in cytokine storm attenuation and EBV-DNA clearance (Fig. 1A). The cytokines IL-10 and IFN-γ levels were significantly decreased within one week (median levels: IL-10: 294.2 pg/mL vs. 6.4 pg/mL, *P* = 0.007; IFN-γ: 77.4 pg/mL vs. 5.1 pg/mL, *P* = 0.009). The other laboratory findings such as hypofibrinogenemia, hyperferritinemia and liver dysfunction were significantly improved when compared with those before treatment. Furthermore, serum EBV DNA levels in 9 children significantly decreased, with 6 children achieving a negative result (viral CR). Overall, the median level of EBV DNA decreased from 1.92 × 10^5^ copies/mL to below detectable levels (< 400 copies/mL).

**Fig. 1:**
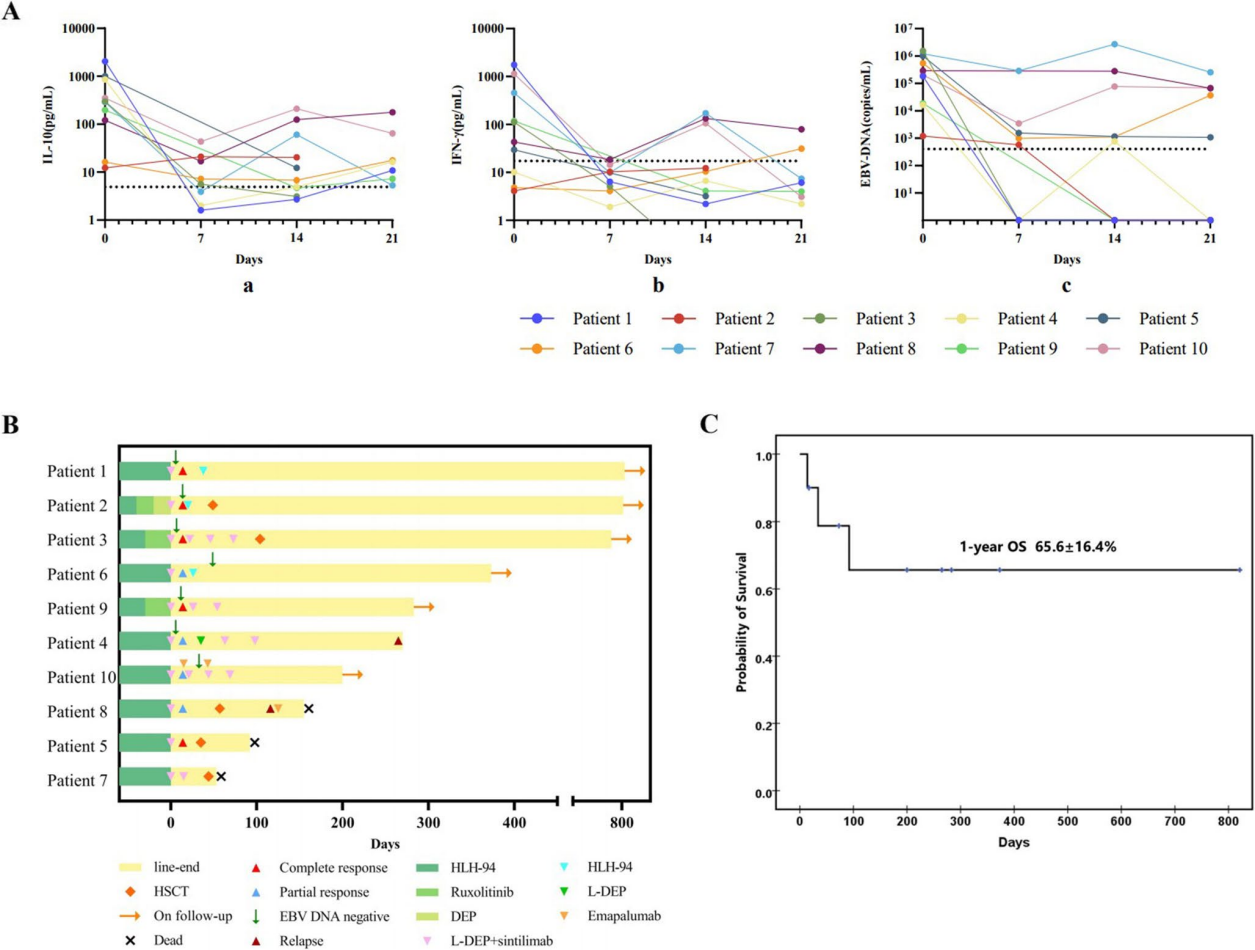
Laboratory findings and clinical outcomes of pediatric patients with R/R EBV-HLH after L-DEP + sintilimab regimens treatment. **A** Changes of IL-10, IFN-γ and serum EBV loads before and after L-DEP + sintilimab regimens treatment. **B** Swimming plot visualizing the response details for L-DEP + sintilimab regimens treatment in 10 patients. **C** Survival status calculated by Kaplan-Meier curve

After one cycle of L-DEP + sintilimab treatment, five children achieved CR, four achieved PR and one remained NR, with an overall response rate of 90% (Fig. 1B). For the five children who achieved CR after one cycle, one continued with the regimen for two more cycles without undergoing HSCT, and has maintained continuous complete remission (CCR) for 9 months without requiring any immunosuppressive therapy. One child was switched to the HLH-94 regimen due to liposomal doxorubicin allergy and is currently in CCR for 27 months, also without any immunosuppressive therapy. Three children underwent HSCT after achieving CR, two of whom are still alive, while one died due to severe gastrointestinal graft-versus-host disease (GVHD). Among the 4 children with PR, one continued with the regimen for two more cycles without undergoing HSCT; however, EBV reactivation and HLH relapse developed after 5 months of treatment. This led to treatment cessation by the patient’s guardians, with subsequent loss to follow-up resulting in right-censoring at the last documented contact. One resumed the HLH-94 regimen for an 8-week course and has maintained complete remission for 12 months, following an initial 2-month period of oral ruxolitinib maintenance therapy which was subsequently discontinued. One achieved CR with the addition of emapalumab to L-DEP + sintilimab regimens, and has been observed for 4 months without relapse. One child who underwent HSCT during the PR phase experienced EBV reactivation 20 days post-transplant and was unable to clear the virus, leading to death 3 months after transplant. The child with NR underwent two additional cycles of L-DEP + sintilimab regimens, achieving PR and subsequently undergoing HSCT, but died due to severe respiratory distress syndrome (RDS) and respiratory failure during granulocyte engraftment. With extended follow-up through June 2025, with a median follow up time of 9 months, the one-year overall survival (OS) rate was 65.6 ± 16.4% (Fig. 1C).

Treatment-related adverse events during PD-1 blockade (sintilimab) plus L-DEP therapy included: myelosuppression (7/10; grade 2: *n* = 5, grade 1: *n* = 2), infectious complications (4/10; all grade 2), hepatic dysfunction (2/10; all grade 2), hypokalemia (2/10; all grade 1), treatment-related allergy (1/10; grade 2). No cytokine release syndrome (CRS) or grade ≥ 3 adverse events were observed. The regimen demonstrated favorable tolerability.

Based on the above results, the L-DEP + sintilimab regimen showed significant symptom relief and viral load decrease in most cases of R/R EBV-HLH. Some patients who achieved satisfying response could survive well without HSCT. In this single-arm pilot study, an EBV clearance rate of 60% was observed with a manageable safety profile (grade ≥ 3 adverse events: 0%), which as much higher than those in clinical studies using L-DEP protocol (virological CR 20%) [1, 2]. However, the potential benefits relative to existing regimens require validation in prospective controlled trials. According to this pilot study, these regimens demonstrate advantages in both efficacy and safety and could serve as a new treatment option for pediatric patients with R/R EBV-HLH or as a bridge to further improve HSCT outcomes.

This exploratory investigation has several key limitations: First, the small number of participants (only 10) makes it hard to draw strong conclusions; Second, the lack of parallel comparator arms prevented causal inference; Third, the follow-up times varied widely, and the final outcome were still uncertain for patients with short follow-up time. This inconsistency makes it difficult to assess long-term effects.

## Data Availability

No datasets were generated or analysed during the current study.
